# Hyaluronic acid as a promoter of the healing of post-extraction socket treated with the socket preservation technique: a systematic review

**DOI:** 10.3389/froh.2025.1583189

**Published:** 2025-05-26

**Authors:** Tommaso Pizzolante, Gianluca Benincasa, Francesco Bizzarro, Mario Capogreco, Enrico Marchetti

**Affiliations:** MESVA, Department of Life, Health & Environmental Sciences, University of L'Aquila, L'Aquila, Italy

**Keywords:** socket preservation, hyaluronic acid, HA, bone graft, ridge preservation

## Abstract

**Background:**

This review examines the role of hyaluronic acid (HA) in enhancing the healing of the post-extraction socket. HA, a naturally occurring glycosaminoglycan in the extracellular matrix, is crucial for wound healing. It promotes tissue repair by stimulating cell migration, adhesion, and proliferation, essential for bone formation. When combined with deproteinized bovine bone mineral (DBBM), HA may improve bone regeneration and reduce resorption, though evidence is still limited.

**Methods:**

Three clinical studies were reviewed, assessing primary outcomes such as volumetric bone resorption, linear bone loss, and soft tissue healing. Radiographic evaluations, including cone beam computed tomography (CBCT), were used to quantify bone changes, while clinical assessments were conducted to evaluate soft tissue responses and wound healing over a 4-month follow-up period.

**Results:**

Two studies demonstrated that HA, in combination with DBBM, significantly improved bone preservation. These studies found reduced volumetric bone resorption and enhanced bone width retention, with one showing a significant reduction in crestal bone loss (*p* < 0.001). In contrast, the third study did not report any significant improvements in soft tissue healing or bone preservation with HA treatment.

**Conclusions:**

The results of this review suggest that HA, combined with DBBM, may offer significant benefits in reducing bone resorption and preserving bone width in ARP procedures. However, the impact of HA on soft tissue healing cannot yet be statistically evaluated, highlighting the need for further investigation to optimize its use in clinical practice.

**Systematic Review Registration:**

https://www.crd.york.ac.uk/PROSPERO/view/CRD42024526628, PROSPERO CRD42024526628.

## 1 Introduction

The loss of a tooth due to disease or trauma is still a common reason for the need to place a prosthetic element. The preservation of alveolar bone following tooth extraction is a critical factor that influences the success in case of an implant placement (1, 2). The morphology of the teeth, their axis of eruption, and their potential inclination determine the shape and volume of the alveolar process itself. Conversely, following tooth extraction, the alveolar process undergoes atrophy, in which the bundle bone at the site loses its function and resorbs (3, 4).

This means that the resorption of post-extraction sockets, without considering the potential damage to the bone tissue during the extraction itself (5), can compromise the volume and height of the alveolar ridge, making it difficult to place implants that are both functional and aesthetically pleasing (6, 7). Various surgical techniques (8) and materials have been developed to address this issue: among these, the Socket Preservation Technique (SPT) has gained significant attention. This technique aims to minimize bone loss by maintaining the structural integrity of the alveolar ridge and enhancing the healing process after tooth extraction (9–12): it can involve autogenous bone, biological agents, or graft materials to limit bone resorption, although they do not eliminate it completely. The results vary depending on the technique used, and the quality of the new bone depends on the type of material employed, which could promote bone regeneration due to its osteoconductive properties (13).

Hyaluronic acid (HA), a naturally occurring glycosaminoglycan found in the extracellular matrix, has increasingly been recognized for its role in wound healing and tissue regeneration (14). HA plays a crucial role in various physiological processes, including cell migration, proliferation, and hydration, which are essential for effective tissue repair (15–18). Hyaluronic acid is a promising biomaterial due to its viscoelastic properties and ability to retain large amounts of water. This makes it an effective periodontal filler and a protective barrier against bacteria and viruses (19–22). Due to its unique properties, HA has emerged as a promising adjunctive treatment in dental procedures from the treatment of oral ulcers to facial aesthetics, orthodontics, periodontal therapy (2, 17, 23), oral surgery, particularly in association with collagen membranes (19, 20) or in combination with bone graft materials such as deproteinized bovine bone mineral (DBBM) (24). The potential synergistic effects of HA and DBBM may enhance bone regeneration and reduce resorption rates in the context of socket (12, 25–28).

The application of HA in socket preservation protocols (7) is supported by its biocompatibility and its ability to promote osteogenesis (24). Research has shown that HA can stimulate the activity of osteoblasts, promote blood vessel formation, and facilitate the integration of graft materials into the surrounding bone (29, 30). These biological properties contribute to improved healing outcomes and may lead to more predictable results in implant placement following socket preservation procedures (5, 31).

Despite the promising potential of HA in enhancing bone regeneration, the existing literature remains limited and often inconclusive. Variability in study designs, methodologies, and outcome measures has hindered the ability to draw definitive conclusions regarding the effectiveness of HA in socket preservation techniques (17, 32). Therefore, this systematic review aims to evaluate all available clinical evidence on the impact of HA on the healing of post-extraction sockets treated with socket preservation techniques. By synthesizing the results of randomized controlled trials, this review seeks to clarify the role of HA in bone preservation, assess its effectiveness compared to conventional treatments, and identify areas for future research.

Understanding the benefits and limitations of HA in socket preservation should not only help clinicians optimizing their treatment protocols but also contribute to advancing the field of regenerative dentistry. The integration of HA into routine clinical practice has the potential to improve patient outcomes and enhance the success of dental implants, making this an important area of investigation (2, 6, 10).

The combination of HA and xenotransplants has achieved numerous successes in animal models over the years (33, 34). Only more recently have RCTs been conducted on humans. While there are currently other recent reviews (35) on the subject in the literature, the presence of some inconsistencies has led the authors of the present review to persist in drafting this one.

By addressing the gaps in knowledge and clarifying the clinical implications of HA in socket preservation, this systematic review has the primary objective of providing a comprehensive understanding of its role in both promoting better healing and reducing resorption of post-extraction sockets by comparing volumetric bone variation between baseline and the end of the follow-up.

## 2 Materials and methods

### 2.1 Study protocol and registration

The protocol was developed according to the International Prospective Register of Systematic Reviews (PROSPERO) guidelines and registered on the platform as CRD42024526628. The systematic review was conducted in accordance with the Cochrane Handbook for Systematic Reviews of Interventions (36).

### 2.2 Information sources, literature search, and eligibility criteria

Articles concerning the use of hyaluronic acid in conjunction with socket preservation techniques were searched in the Medline, Scopus, and Web of Science databases. The search strategy was limited to randomized controlled trials (RCTs) focused on bone preservation utilizing hyaluronic acid (HA) with socket preservation techniques. The eligibility criteria included studies conducted on human subjects with compromised teeth requiring extraction followed by an alveolar ridge preservation technique, in which hyaluronic acid was used in combination with xenografts. Patients had to be aged ≥ 18 years and non-smokers. Excluded from this review were: studies on animal models, reviews, studies not involving socket preservation techniques, studies not involving hyaluronic acid with deproteinized bovine bone mineral (DBBM) or other xenografts, non-randomized studies and *in vitro* studies.

The search strategy was:

- PubMed:
  • [“Alveolar Ridge Preservation”[All Fields] OR “Socket Preservation”[All Fields]] AND [“hyaluronic acid”[MeSH Terms] OR “HA"[All Fields] OR “hyaluronic acid”[All Fields]];
  • [“Alveolar Ridge Preservation”[All Fields] OR “Socket Preservation”[All Fields]] AND [“hyaluronic acid”[MeSH] OR “HA”[All Fields] OR “hyaluronic acid”[All Fields]] AND “Bone Graft” [All Fields].
- Other Platforms and Database:
  • (“Hyaluronic Acid” OR “HA”) AND (“Ridge Preservation” OR “Socket Preservation” OR “Alveolar Ridge Preservation”);
  • (“Hyaluronic Acid” OR “HA”) AND (“Ridge Preservation” OR “Socket Preservation” OR “Alveolar Ridge Preservation”) AND (“Bone Graft”).

**Figure 1:**
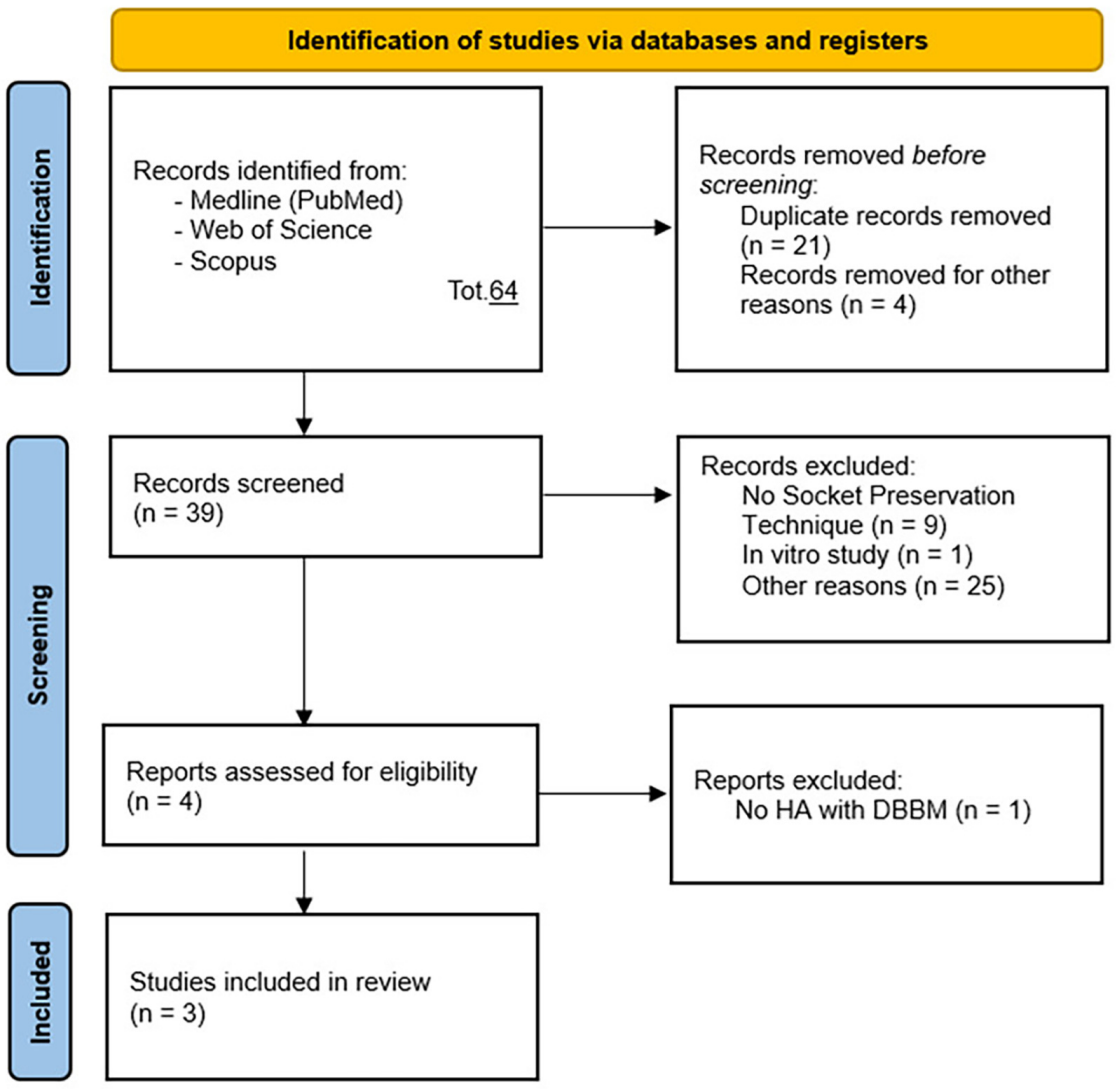
PRISMA 2020 flow diagram (48) for new systematic reviews which included searches of databases and registers only.

Articles were initially selected based on titles and abstracts (Figure 1), followed by full-text analysis, resulting in a table that included: author, year, title, type of article, population, outcomes, biases, inclusion, and exclusion criteria. Then documents were selected based on the following Population, Intervention, Control, and Outcome (PICO) model:

- Population: Human subjects, aged at least ≥18, who need Tooth extraction;
- Intervention: Tooth extraction with Socket Preservation or Alveolar Ridge Preservation Techniques combined with Hyaluronic Acid;
- Control: Tooth extraction with Socket Preservation or Alveolar Ridge Preservation Techniques without Hyaluronic Acid;
- Outcome: Efficacy of combination of Hyaluronic Acid with Socket or Alveolar Ridge Preservation Techniques in reducing bone resorption after Tooth extraction.

Two operators conducted the research, initially including previous systematic reviews related to the topic in question. After concluding that there were inconsistencies in the previously conducted reviews regarding the mismatch between the stated primary objective and the included studies, the research continued in accordance with PRISMA guidelines. Two operators searched and collected studies. As shown in Figure 1, a total of 64 potential references were identified, and after removing duplicates, 39 studies remained for title and abstract selection. 37 studies were excluded for not meeting the inclusion criteria of this systematic review: *n* = 9 for not employing socket preservation techniques, *n* = 5 as preclinical animal studies, *n* = 2 as reviews, *n* = 1 as a preclinical *in vitro* study, *n* = 1 as a prospective study, *n* = 17 for other reasons. Following careful full-text analysis, one additional study was excluded for not utilizing hyaluronic acid with DBBM nor being an RCT. Thus, this systematic review included 3 randomized controlled clinical studies (RCTs).

### 2.3 Data collection and extraction

Two operators extracted the following information from the studies: (a) first author, (b) year of publication, (c) study design, (d) type of treatment, (e) treatment site, (f) type of HA application, (g) treatment groups, (h) follow-up period, (i) available outcome parameters. Additionally, all available information on HA products was summarized, including (a) trade name, (b) manufacturer, (c) concentration, (d) chemical form. In case of disagreement between the first two operators, the data and their eligibility were discussed with a third operator.

## 3 Results

### 3.1 Description of studies considered

Three studies were considered (Table 1): Eeckhout et al. (37), Husseini et al. (38), Abaza et al. (39). The included studies examined the effectiveness of combining hyaluronic acid (HA) with deproteinized bovine bone mineral (DBBM—Xenogeneic) for socket or alveolar ridge preservation following tooth extraction. Those RCT aimed to evaluate impact of HA on bone regeneration, volumetric bone loss, and graft material integration. Although all of the studies shared similar objectives, their designs and patient cohorts were different, offering complementary perspectives on HA's clinical utility. Eeckhout et al. (37), Husseini et al. (38) focused on alveolar ridge preservation, whereas Abaza et al. (39) focused on socket preservation.

**Table 1:** Description of considered studies.

Name of the study (year)	Design	Participants	Groups and intervention details	Authors’ conclusions
Eeckhout et al. (37)	RCT	38 patients (22 women and 16 men) Scheduled for ARP, with 23 sites for each group (TG and CG)	Group 1 (TG): Sockets treated with HA gel (application of 0.8% HA gel) and C-DBBM. HA gel applied by the patient onto the collagen matrix. Group 2 (CG): Sockets treated with C-DBBM alone.	HA administration does NOT seem to optimize the results of ARP.
Husseini et al. (38)	RCT (*pilot*)	7 patients with a total of 14 hopeless teeth (bilateral teeth in each patient)	Group 1 (TG): Sockets treated with a combination of HA (*mixture of cross-linked HA*) and DBBM. Group 2 (CG): Sockets treated with DBBM alone.	Cross-linked hyaluronic acid (xHyA) appears to limit the post-extractional alveolar bone resorption when mixed with DBBM.
Abaza et al. (39)	RCT	36 patients (20 women and 16 men) requiring implant placement in the anterior maxilla	Group 1 (TG1): Sockets treated with HA combined with DBBM. Group 2 (TG2): Sockets treated with injectable platelet-rich fibrin (I-PRF) combined with DBBM. Group 3 (CG): Sockets treated with DBBM alone, mixed with saline.	The combination of HA with xenografts demonstrated superior outcomes in preserving bone dimensions compared to xenografts alone or combined with I-PRF, both clinically and radiographically.

RCT, randomized controlled trials; TG, test group; CG, Control group; ARP, alveolar ridge preservation; DBBM, deproteinized bovine bone mineral; C-DBBM, collagen-enriched, deproteinized bovine bone mineral.

Husseini et al. (38) conducted a randomized split-mouth pilot study involving seven patients, each with bilateral hopeless teeth, leading to the extraction of 14 teeth in total. After atraumatic extraction, one socket in each patient was treated with a combination of HA and DBBM, while the other socket received only DBBM. The primary outcomes, assessed through cone-beam computed tomography (CBCT) scans and histological biopsies, revealed that sockets treated with HA experienced significantly less bone resorption and improved graft integration compared to those treated with DBBM alone. This indicates HA's beneficial role in preserving alveolar ridge volume and enhancing the integration of graft materials, leading to better preparation for future implant placement.

Abaza et al. (39), on the other hand, conducted a larger randomized controlled trial with 36 patients requiring implant placement in the anterior maxilla. The study included three groups: one treated with HA and DBBM, another with injectable platelet-rich fibrin (I-PRF) and DBBM, and a control group treated with DBBM mixed with saline. Similar to Husseini et al., outcomes were measured using CBCT to assess volumetric bone changes at a four-month follow-up. In addition, histomorphometric analysis was performed to evaluate new bone formation and graft integration. The results showed that the group treated with HA and DBBM had the least bone loss and the best graft integration, outperforming both the I-PRF and DBBM-only groups.

In Eeckhout et al. (37), the study included a 4-month follow-up to assess the effects of HA gel combined with DBBM on alveolar ridge preservation. The primary outcomes focused on changes in wound dimensions (bucco-lingual and mesio-distal measurements) and soft tissue healing at 1 week and 3 weeks after the procedure. Bone resorption was measured through radiographic analysis using CBCT scans, and a significant increase in horizontal bone loss was observed in the HA-treated group compared to the control group (*p* ≤ 0.025). These findings suggest that hyaluronic acid gel did not improve bone preservation or soft tissue healing significantly when compared to the control group treated with DBBM alone.

An important difference, however, concerns the use of hyaluronic acid among the three studies: in the first study [Eeckhout et al. (37)] patients were instructed by the surgeon to apply an HA-based gel during the seven days following the procedure (the first application was shown by the surgeon), whereas in the studies by Husseini et al. (38) and Abaza et al. (39), the HA was applied directly by the surgeon intraoperatively.

The authors also analyzed a fourth study that did not meet the inclusion criteria of this systematic review [Kloss et al. (40)]: it was a retrospective comparative study (not an RCT) that combined hyaluronic acid (HA) with an allograft bone substitute derived from human donor bone and not a xenograft. Although it was not possible to include this study, it has been briefly described in the discussion section to provide an overview of the different possible perspectives and applications of HA. Previous systematic reviews with a primary objective similar to the present one, particularly regarding the use of xenografts, have included this study, despite the clear specification of the use of allografts.

### 3.2 Risk of bias assessment

**Figure 2:**
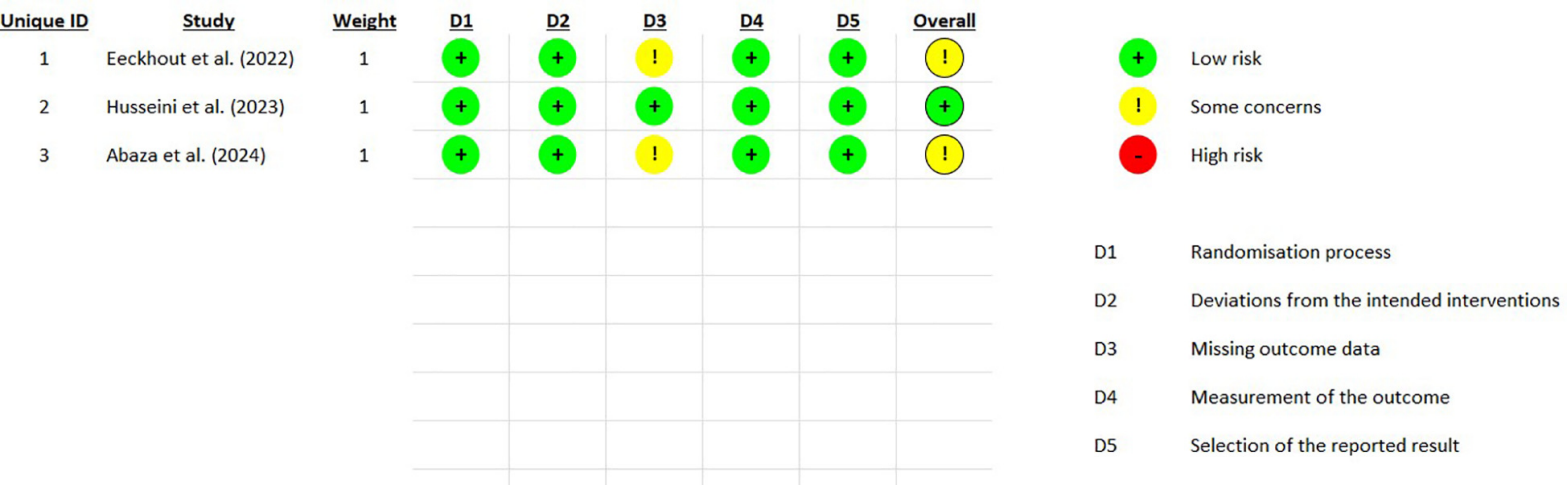
Traffic light plot (RoB).

The Cochrane Collaboration's RoB2 RPG V9 tool (41) was utilized for the assessment of bias risk. The following domains were evaluated, each assigned a Risk of Bias (RoB) rating of “low”, “medium”, or “high” (Figure 2):

1. Randomization process;
2. Deviations from intended interventions;
3. Missing data;
4. Measurement of outcomes;
5. Selection of reported outcomes.

The overall RoB calculated by the algorithm was rated as *medium* for two studies [Eeckhout et al. (37) and Abaza et al. (39)] and as *low* for the third study [Husseini et al. (38)].

### 3.3 Interventions in studies and HA information

In the studies examined [Table 2 (37–39)] hyaluronic acid was applied following tooth extraction. In Eeckhout et al. (37) patients were scheduled for alveolar ridge preservation (ARP) following tooth extraction at one or two sites in the aesthetic zone (incisor, cuspid, or premolar area). After local anesthesia, the teeth were extracted atraumatically to preserve the surrounding bone. The experimental group (TG) received hyaluronic acid gel (0.8%) applied on a collagen matrix (Mucograft® Seal, Geistlich Pharma AG, Switzerland) following deproteinized bovine bone mineral (DBBM, Bio-Oss®, Geistlich Pharma AG, Switzerland) filling in the socket. In the control group (CG), only DBBM was placed in the socket. No gingival grafts were used. After 1 week, sutures were removed, and patients were followed for up to 4 months. Primary outcomes were changes in wound dimensions (bucco-lingual and mesio-distal) at 1 week and 3 weeks post-surgery. Secondary outcomes included analgesic consumption, pain levels, and alveolitis incidence. Histomorphometric outcomes (for example new bone formation) were not directly measured, and bone resorption was quantified radiographically (in width). At 4 months, no significant differences were found between groups regarding soft tissue healing or wound dimensions. However, a significant increase in horizontal bone loss was observed in the test group treated with hyaluronic acid gel (*p* ≤ 0.025). These findings suggest that hyaluronic acid did not improve wound healing or bone preservation following alveolar ridge preservation and may have led to more bone resorption at the coronal aspect.

**Table 2:** Procedures performed in the analyzed studies.

Analyzed elements	Name of the study (year)		
	Eeckhout et al. (2022) (37)	Husseini et al. (2023) (38)	Abaza et al. (2024) (39)
Bone materials used	Deproteinized bovine bone enriched with collagen^b^ (Bio-Oss Collagen®)	Deproteinized bovine bone^a^, without collagen (Bio-Oss®)	Deproteinized bovine bone^a^ (Cerabone®)
Solutions/additional materials	HA gel 0.8% (Gengigel Forte®), (only in the TG).	– xHyA (cross-linked HA, Hyadent BG®): TG. Epithelial-connective tissue punches to seal the socket.	– Cross-linked HA (20 mg/ml): TG1. – I-PRF (injectable platelet-rich fibrin): TG2. – Saline solution (CG).
Surgical techniques	– Extraction without flap elevation (papillary incisions if needed). – Protection with collagen matrix (Mucograft Seal®).	– Atraumatic extraction preserving the buccal plate. – Closure with epithelial-connective tissue punches.	– Socket filled with sticky bone or putty. – Closure with a free gingival flap (2 mm) and Teflon template.
Post-operative treatment (not considering antibiotic pre-medication)	– Antibiotics: Amoxicillin 2 g/day for 4 days. – Anti-inflammatories: Ibuprofen (600 mg) as deemed necessary by the patient.	– Antibiotics: Amoxicillin 2 g/day for 7 days. – Anti-inflammatories: Ibuprofen 400 mg for 3 days. – Mouthwash: Chlorhexidine 0.12% for 2 weeks.	– Antibiotics: Amoxicillin 875 mg and clavulanic acid 125 mg twice daily for 5 days. – Mouthwash: Chlorhexidine 0.12% for 7 days.
Study variables	Evaluation of the efficacy of HA gel 0.8% compared to C-DBBM alone.	Comparison between pure DBBM and DBBM + xHyA, focusing on the healing of soft and hard tissues.	Comparison of: – I-PRF, – Cross-linked HA, – Saline solution with Cerabone®.
HA application timing and operator	After surgery First application: *surgeon*, later applied by *patient* for 7 days.	Intraoperative Mixed with bone material by the *surgeon* before socket filling.	Intraoperative Mixed with bone material by the *surgeon* before socket filling.

TG, test group; CG, control group.

^a^ DBBM, deproteinized bovine bone mineral.

^b^ C-DBBM, collagen-enriched, deproteinized bovine bone mineral.

In Husseini et al. (38), following local anesthesia (Septanest, Septodont, Saint Maur des Fosses, France), teeth were extracted atraumatically with an effort to preserve the vestibular bone and were curetted under abundant saline irrigation using a Lucas curette (Hu Friedy, CHI, USA). The control site received only DBBM (Bio-Oss, Geistlich Pharma AG, Switzerland), while the experimental site was filled with a mixture of DBBM and xHyA (Hyadent BG, Regedent AG, Switzerland). Depending on the diameter of the socket, two pieces of connective tissue from the palate were harvested to seal the socket. Finally, a collagen membrane (CollaTape, Zimmer Biomet, USA) was sutured at the donor site using resorbable suture material 4.0 (Novosym, B-Braun, Melsungen, Germany) for site protection.

In Abaza et al. (39), local anesthesia was administered via buccal and palatal infiltration using mepivacaine (2%) with levonordefrin (1:20,000) (Mepecaine-L, Alexandria, Egypt) as a vasoconstrictor. The extraction procedure was performed using periosteo elevators and forceps, employing an atraumatic technique to preserve surrounding bone. After extraction, the socket was carefully curetted to ensure the removal of any residual soft tissue. The socket was then irrigated with saline to maintain cleanliness. In group 1, I-PRF preparation followed the method described by Wang et al. in 2017 (32). Venous blood samples (10 ml) were collected without anticoagulants and centrifuged in a plastic tube at 700 rpm for 3 min (32). The resulting I-PRF was mixed with DBBM (Cerabone®, Straumann, Germany) to create a sticky bone consistency. This mixture was carefully placed into the socket to fill it completely up to the gingival margin. In group 2, a syringe containing 1 ml of cross-linked HA (xHyA) solution at a concentration of 20 mg/ml (Perfectha, France) was mixed with DBBM in a 1:10 ratio, resulting in a putty-like consistency. The HA-DBBM putty was condensed and carefully placed in the post-extraction socket up to the gingival margin. In group 3, DBBM was mixed with saline. The mixture was then inserted into the socket up to the gingival margin, serving as the positive control group in the study. To achieve socket sealing, a free gingival graft of approximately 2 mm thickness was harvested from the palate. The socket was sutured using cross stitches with 4-0 polypropylene sutures (Oralsply, Dtek, Taoyuan, Taiwan), providing stability to the graft material. After a 4-month healing period, a re-entry procedure was performed. The mucoperiosteal flaps were elevated to provide access to the alveolar ridges of the affected sockets via a horizontal crestal incision without vertical incisions. A 2.5 mm diameter bone biopsy sample was obtained, and implants (Dentium SuperLine II, Gangan-gu, Seoul, South Korea) were placed with submerged healing. The overlying flap was sutured with a tension-free closure achieved using simple interrupted sutures in 5.0 polypropylene (Oralsply, Dtek, Taoyuan, Taiwan). Patients were recalled 6 months after implant placement for the definitive crown placement.

### 3.4 Reported outcome variables and follow-up

All clinical studies considered evaluated volumetric changes at the sites via radiographic examination. The study by Abaza et al. (39) assessed volumetric changes at 4 months using CBCT radiographic examination, with the primary outcome being the radiographic width of bone measured using CBCT before surgery and 4 months after alveolar augmentation. It is important to note that, although the secondary outcomes of Abaza et al. (39) were measured with follow-up up to one year after surgery, the primary outcomes were only measured at 4 months. A fusion technique was employed to evaluate changes in both vertical and horizontal dimensions of the alveolar ridge. Initially, preoperative and 4-month postoperative CBCT scans were superimposed. Subsequently, multiple anatomical landmarks were traced, and a line dividing the anatomical site boundary was drawn, serving as a reference for assessing changes in the alveolar ridge. Buccolingual widths were measured at various levels:

1. On the bony crest;
2. mm from the bony crest;
3. 5 mm from the bony crest.

Radiographic assessment for estimating volumetric changes in the alveolar ridge in the Husseini et al. (38) study was conducted by overlaying pre- and postoperative CBCT scans (i-CAT!, Hatfield, PA, USA) using semi-automatic contour segmentation software (ITK-SNAP). Volumetric changes were assessed in both groups through the software. Patients were recalled after 4 months for further evaluation of the bone's structural properties through radiographic examination.

In Eeckhout et al. (37), volumetric changes were also evaluated using CBCT (cone beam computed tomography) at the 4-month follow-up. The primary outcome was the horizontal and vertical bone loss following alveolar ridge preservation (ARP) using DBBM and HA gel (0.8%). The control group (CG) received only DBBM, while the experimental group (TG) received DBBM in combination with hyaluronic acid gel and collagen matrix. The CBCT images were superimposed pre- and postoperatively. Radiographic analysis showed that both groups experienced significant horizontal resorption at the coronal aspect, with the test group (TG, treated with HA gel) demonstrating greater horizontal bone loss compared to the control group (CG) (*p* ≤ 0.025). These findings suggest that hyaluronic acid did not improve wound healing or bone preservation following alveolar ridge preservation and may have led to more bone resorption at the coronal aspect.

### 3.5 Outcomes of included studies

All of the considered studies (37–39) assessed volumetric changes as one of their outcomes, with results determined through radiographic analysis using CBCT. However, the primary outcomes varied among studies:

• Eeckhout et al. (37): The primary outcomes were wound dimensions (bucco-lingual and mesio-distal changes) and soft tissue healing at 1 and 3 weeks post-procedure. The evaluations related to the bone condition were part of the secondary outcomes. HA did not significantly improve wound resolution or reduce horizontal bone loss compared to control (*p* > 0.05). Secondary outcomes included soft tissue healing and the incidence of alveolitis, with no significant differences between groups.
• Husseini et al. (38): This study primarily focused on volumetric and linear bone resorption. The addition of cross-linked HA (xHyA) to DBBM significantly reduced both volumetric bone resorption [26.96% (TG) vs. 36.56% (CG)] and linear bone resorption (0.73 mm vs. 1.42 mm) compared to DBBM alone (*p* = 0.018).
• Abaza et al. (39): The primary outcome was residual bone width, where HA proved more effective than I-PRF or control (DBBM with saline). The mean residual bone width was 9.78 ± 0.87 mm in the HA group (TG1), compared to 8.60 ± 1.27 mm in the I-PRF group (TG2) and 7.99 ± 0.89 mm in the control group (*p* = 0.007).

Additionally, Abaza et al. (39) reported a significant reduction in crestal bone loss in the HA group compared to control (*p* < 0.001). These data are shown in Table 3, while secondary outcomes are reported in Table 4.

**Table 3:** Results of the studies.

Study	Follow-up	Groups	Volumetric bone resorption (in width—at level 1—at 4 months)		Crestal bone loss	Histomorphometric outcomes
Eeckhout et al. (37)	4 months	TG: (DBBM + HA gel)	3.55 mm ± 0.87 mm (*p*-value: ≤ 0.025)		BH: 1.00 ± 0.32 mm (*p* = 0.237); LH: 1.46 ± 0.45 mm (*p* = 0.351)	No % of new bone reported (measurement at 1, 3 and 5 mm in the main text) - Residual graft material: not reported
		CG: (DBBM + saline solution)	1.92 mm ± 0.65 mm (*p*-value: ≤ 0.025)		BH: 0.45 ± 0.17 mm (*p* = 0.237); LH: 0.96 ± 0.38 mm (*p* = 0.351)	No % of new bone reported (measurement at 1, 3 and 5 mm in the main text) - Residual graft material: not reported
Study	Follow-up	Groups	Volumetric bone resorption (% at 4 months)		Crestal bone loss	Histomorphometric outcomes
Husseini et al. (38)	4 months	TG (DBBM + HA)	26.96 ± 1.83% (*p* = 0.018)		0.73 ± 0.052 mm (*p* = 0.018)	Test sites showed greater incorporation of DBBM into newly formed bone with better graft integration.
		CG (DBBM alone)	36.56 ± 1.69% (*p* = 0.018)		1.42 ± 0.16 mm (*p* = 0.018)	Control sites showed DBBM embedded in connective tissue, indicating poorer graft integration.
Study	Follow-up	Groups	Radiographic bone width		Crestal bone loss	Histomorphometric Outcomes
			Baseline	At 4 months		
Abaza et al. (39)	4 months	TG1: (DBBM + HA)	9.96. ± 2.45	9.78 ± 0.87 mm (highest bone width—*p* = 0.007)	0.33 ± 0.15 mm (lowest loss—*p* < 0.001)	56.66 ± 7.35% new bone (highest) - Residual graft material: 2.63 ± 1.27% (lowest)
		TG2: (DBBM + I-PRF)	9.23 ± 2.39	8.60 ± 1.27 mm (*p* = 0.007)	0.53 ± 0.11 mm (*p* < 0.001)	28.74 ± 5.15% - Residual graft material: 6.76 ± 2.59%
		CG: (DBBM)	9.26 ± 0.99	7.99 ± 0.89	0.98 ± 0.18	24.05 ± 3.64 - Residual graft material: 2.71 ± 1.24%

TG, test group; CG, control group; BH, bone height at the buccal aspect; LH, bone height at the lingual aspect.

**Table 4:** Secondary outcomes.

Study	Secondary outcomes	Main findings
Eeckhout et al. (37)	Secondary outcomes registered in the early healing phase	Soft tissue healing (measured with SWHS) and the incidence of alveolitis, with no significant differences between groups.
	Secondary outcomes registered at 4 months	Changes in bone dimensions: More horizontal shrinkage in the test group
		Soft tissue changes: NO significant differences regarding soft tissue profile and height in different points
		Mucosal scarring index: NO significant difference
Husseini et al. (38)	NO secondary outcomes clearly specified	-
Abaza et al. (39)	Soft tissue thickness	No significant difference was observed between the different groups at the preoperative, 4-month, and 1-year postoperative time points. When comparing the mean difference at 1 year postoperative to the preoperative measurements, the I-PRF group exhibited the highest value and a greater long-term stability.

SWHS, socket wound healing score (49).

## 4 Discussion

In this systematic review, the combination of hyaluronic acid (HA) and deproteinized bovine bone mineral (DBBM) (42) has been shown to reduce alveolar bone resorption and improve bone quality in post-extraction sites. The studies by Eeckhout et al. (37), Husseini et al. (38) and Abaza et al. (39) provide contrasting evidence regarding the effectiveness of HA in socket preservation procedures.

The results from Husseini et al. (38) and Abaza et al. (39) support the use of HA as an adjunctive treatment, demonstrating that the addition of HA to DBBM leads to improved clinical outcomes, such as better preservation of the alveolar ridge and increased success rates in subsequent implant placements. These results were summarized using the symbols “+” (indicating treatment efficacy) and “-” (indicating lack of treatment efficacy): overall, two “+” and one “-” were obtained. This factor holds limited significance due to the small number of studies available (a number reduced because of the limited literature on the use of HA in post-extraction socket preservation practices).

Husseini et al. (38), in a randomized split-mouth pilot study, found a significant reduction in bone resorption and an improvement in graft integration, highlighting HA's role in maintaining the structural integrity of the alveolar ridge. Abaza et al. (39), with a larger randomized controlled trial, confirmed that HA, when used in combination with DBBM, resulted in better bone preservation compared to DBBM alone, showing superior results even compared to I-PRF. On the other hand, as shown in Table 4, in Abaza et al. (39), measurements of soft tissue thickness taken one year postoperatively, compared to preoperative values, showed that the I-PRF group exhibited the highest values and greater long-term stability. Platelet-rich fibrin (PRF), which belongs to the second generation of blood concentrates (43), is prepared through a single-step centrifugation process without the use of any anticoagulants (44). PRF consists of platelets, leukocytes and their subgroups embedded in a fibrin matrix with plasma proteins. The centrifugation process activates coagulation, thereby promoting clot formation. This clot is made up of a three-dimensional fibrin network in which platelets and other blood cells are trapped (43). Although various protocols have been described in the literature, the Injectable-PRF used in the study by Abaza et al. (39), introduced in early 2017 by Choukroun and Ghanaati (43), was obtained following the protocol described by Wang et al. in 2017 (32). Twenty-eight venous blood samples (10 ml each) were collected from the patient without anticoagulants. The blood samples were then centrifuged in a glass-coated plastic tube at 700 revolutions per minute for 3 min. According to recent systematic reviews (45, 46), PRF has proven effective in reducing postoperative pain, accelerating soft tissue healing, and preventing dimensional bone loss, especially during the initial 2–3 month period, and it showed significant results in all three outcomes when compared to no grafting at all, even if the effect was smaller when compared to other commonly used grafting materials. Although the present systematic review focused on the effectiveness of HA in terms of healing and the reduction of post-extraction bone resorption, it is important to highlight that PRF also represents a promising approach.

On the other hand, Eeckhout et al. (37), despite observing positive effects of HA on soft tissue healing and wound size, did not report significant improvements in bone preservation or graft integration, suggesting that HA had no substantial effect on the final outcome in terms of bone regeneration, raising questions about the efficacy of HA in the absence of synergistic effects with other biomaterials. However, the method of HA application should be interpreted with caution as a determining factor in the results obtained: this method differs in Eeckhout et al. (37) compared to the other two studies. As noted in Table 3, while the first two studies applied HA intraoperatively directly to the post-operative wound, in Eeckhout et al. (37) HA was placed in contact with the collagen matrix and soft tissues, not directly in contact with the heterologous bone graft. Moreover it should still be considered that in Eeckhout et al. (37), although HA gel was applied over the surgical wound for the first 7 days (post-surgery), only the first application was performed by the surgeon, while the subsequent applications were carried out by the patients themselves.

In general, the results of Husseini et al. (38) and Abaza et al. (39) suggest that HA may improve bone preservation, while Eeckhout et al. (37) showed no significant improvements. Although the four-month follow-up provided sufficient data to observe early healing signs, none of the studies provided long-term data, which is crucial to confirm the stability of the regenerated bone and the success of subsequent implant placement. Another important difference to consider in the overall comparison of results is related to the material used for the heterologous bone graft: although all three studies use deproteinized bovine bone, these belong to different lines and/or manufacturers; Eeckhout et al. (28) used Deproteinized Bovine Bone enriched with Collagen, while Husseini et al. (27) and Abaza et al. (26) used Deproteinized Bovine Bone without Collagen. Although this systematic review did not aim to evaluate which type of DBBM yielded the best results (given the limited number of studies that could be considered), this factor should still be taken into account.

Another difference among the studies considered lies in the use of cross-linked hyaluronic acid (xHyA) by Husseini et al. (38) and Abaza et al. (39), whereas Eeckhout et al. (37) used linear hyaluronic acid. While cross-linked HA offers greater structural stability and prolonged tissue residence time, linear HA is more bioactive in stimulating fibroblast proliferation, particularly in the early phases of tissue healing (47).

Based on the studies analyzed, the observed bone resorption seems to be primarily linked to the absence of bioactive materials rather than the morphology of the defect. In the randomized clinical trial by Abaza et al. (39), the groups treated with HA or I-PRF in combination with xenograft showed a significant reduction in crestal resorption and greater new bone formation compared to the control group (xenograft alone), suggesting that the absence of biological stimulators leads to greater bone resorption. These results are also confirmed by Husseini et al. (38), where histomorphometric analysis also highlights reduced integration of the graft in the control group. In contrast, in the study by Eeckhout et al. (37), where HA was applied on a collagen matrix, no significant differences were observed compared to the control, with even greater horizontal bone loss in the HA group, indicating once again that the effectiveness of HA may depend on how it is used.

Another study, Kloss et al. (40), a randomized controlled trial which investigated the effects of HA combined with allogeneic bone graft material for socket preservation, was excluded from this systematic review because it was a retrospective comparative study and also because of the origin of the graft. Anyway, this study (40) involved patients requiring tooth extractions followed by ridge preservation procedures. Unlike the previous studies, which used Xenografts, this study utilized an allogeneic bone substitute derived from human donor bone, combined with HA to evaluate bone regeneration and volumetric stability. The study assessed outcomes at different time points using CBCT imaging and histological analysis. Results (40) demonstrated that the combination of HA and allograft material facilitated significant bone regeneration. Previous systematic reviews with a primary objective similar to the present one, particularly regarding the use of xenografts, have included this study, despite the clear specification of the use of allografts.

Clinical implications suggest that HA, in combination with DBBM, represents a promising strategy to improve bone preservation. However, the high heterogeneity of the studies prevented a meta-analysis of the data, which would have enabled an overall evaluation with statistical significance. To this end, larger studies with longer follow-up periods are necessary to confirm these results, in order to later, if the protocol proves effective, establish standardized protocols for the use of HA in socket preservation techniques. Another parameter that should ultimately be considered in future studies is the impact of using hyaluronic acid on the costs of socket preservation procedures.

## 5 Conclusions

The combination of Hyaluronic Acid (HA) with Deproteinized Bovine Bone Mineral (DBBM) shows significant potential for improving clinical outcomes in socket preservation, particularly in terms of reducing bone resorption and enhancing graft integration. As a result, the integration of HA into routine clinical practice may contribute to higher success rates in dental implantology, making it a valuable tool in the field of regenerative dentistry. Further RCT with longer follow-ups and larger samples are needed to make a meta-analysis of data proving a statistical significance of the efficacy of the treatment and to refine treatment protocols and fully understand the long-term benefits of HA in socket preservation. The use of collagen-free DBBM could also allow for better standardization of future protocols, thus reducing the heterogeneity of the materials used and making the data comparable.

As highlighted by the present systematic review, in order to enable a quantitative analysis of future study outcomes, it would be advisable to assess standardized parameters. In this regard, careful evaluation of bone resorption between baseline and subsequent follow-up periods across different groups is recommended, along with attention to changes in soft tissue conditions. In conclusion, a more thorough assessment of the cost–benefit ratio of incorporating hyaluronic acid into post-extraction socket preservation protocols is warranted, considering biological efficacy, economic impact, and practical applicability in routine clinical practice.

## Data Availability

The original contributions presented in the study are included in the article/Supplementary Material, further inquiries can be directed to the corresponding author.
